# Public attitudes toward genetic modification in dairy cattle

**DOI:** 10.1371/journal.pone.0225372

**Published:** 2019-12-02

**Authors:** Caroline Ritter, Adam Shriver, Emilie McConnachie, Jesse Robbins, Marina A. G. von Keyserlingk, Daniel M. Weary

**Affiliations:** Animal Welfare Program, Faculty of Land and Food Systems, University of British Columbia, Vancouver, British Columbia, Canada; University of Florida, UNITED STATES

## Abstract

Genetic modification has been used to create dairy cattle without horns and with increased resistance to disease; applications that could be beneficial for animal welfare, farm profits, and worker safety. Our aim was to assess how different stated purposes were associated with public attitudes toward these two applications using a mixed methods approach. Using an online survey, U.S. participants were randomly assigned to one of ten treatments in a 2 (application: hornless or disease-resistant) x 5 (purposes: improved animal welfare, reduced costs, increased worker safety, all three purposes, or no purpose) factorial design. Each participant was asked to read a short description of the assigned treatment (e.g. hornlessness to improve calf welfare) and then respond to a series of questions designed to assess attitude toward the treatment using 7-point Likert scales (1 = *most negative*; 7 = *most positive*). Responses of 957 participants were averaged to creative an attitude construct score. Participants were also asked to explain their response to the treatment. Qualitative analysis of these text responses was used to identify themes associated with the participants’ reasoning. Participant attitudes were more favorable to disease resistance than to hornlessness (mean ± SE attitude score: 4.5 ± 0.15 vs. 3.7 ± 0.14). In the ‘disease-resistance’ group participants had more positive attitudes toward genetic modification when the described purpose was animal welfare versus reduction of costs (contrast = 1.00; 95% CI = 0.12–1.88). Attitudes were less favorable to the ‘hornless’ application if no purpose was provided versus when the stated purpose was either to improve animal welfare (contrast = 0.95; 95% CI = 0.26–1.64) or when all purposes were provided (contrast = 0.88; 95% CI = 0.19–1.58). Similarly, attitudes were less positive when the stated purpose was to reduce costs versus either improving animal welfare (contrast = 0.86; 95% CI = 0.09–1.64) or when all purposes were provided (contrast = 0.79; 95% CI = 0.02–1.56). Quantitative and qualitative analysis indicated that both the specific application and perceived purpose (particularly when related to animal welfare) can affect public attitudes toward genetic modification.

## Introduction

Genetic modification (GM) technology has been applied to agricultural crops to improve disease and herbicide resistance, increase resilience to abiotic stress (e.g. heat and drought), lengthen shelf life, and increase yield (see [1]). GM technology also has the potential to improve production efficiency, nutritional value of food products, health, and welfare of agricultural animals [see 2]. However, there is currently only one GM animal approved for human consumption in the United States (AquAdvantage salmon [3]) and although various GM animals have been developed for use in laboratories (see [4]), the idea remains controversial [5].

Recently, scientists have genetically modified dairy cattle to be hornless and to be resistant to certain diseases [6–8]. The hornless phenotype occurs naturally in some cattle breeds and is controlled by a single dominant gene (*POLLED*). Using CRISPR technology, it is possible to integrate this gene into the genome of other cows [6] thus eliminating the need for surgically removing horns or horn buds. Dehorning (or disbudding) is a common surgical procedure, typically performed without pain mitigation on U.S. dairy farms [9–11]. Thus, producing genetically hornless cattle could reduce the suffering of animals. In a similar vein, inducing disease resistance through GM poses an opportunity to improve animal health and welfare. For example, GM cattle have been developed that are resistant to mastitis [7] and bovine tuberculosis [8]. Mastitis is a painful inflammation of the udder experienced by approximately 25% of U.S. dairy cows each year [12] and bovine tuberculosis is a contagious disease that poses a threat to humans and other species [13].

Besides the potential to improve animal welfare, GM hornless and disease resistant cattle may also provide financial benefits to farmers and others involved in livestock production. For example, farmers may benefit from hornless cattle by saving costs associated with dehorning and from disease resistant cattle by reducing medical expenses and production losses. Additionally, these GM cattle may benefit farm workers. For example, workers benefit from hornless cattle by not having to conduct dehorning surgery or work with horned cattle, and in the case of disease resistance may benefit from having less work, more pleasant working conditions, and being at lower risk of acquiring a zoonotic disease.

Despite these potential benefits, GM animals are often perceived negatively by the public, although US citizens are generally less critical than Europeans [14]. Furthermore, intention to purchase GM products derived from animals is generally lower than the intention to purchase products developed from GM plants [14]. This negative perception may help explain why few foods derived from GM animals have entered the food system, as public opinion influences policymakers, research funding, and markets [15]. Perceived risks, benefits, naturalness, and trust are among the factors that influence public attitudes toward genetically modified products [e.g. 16–24]. If tangible benefits are perceived for specific applications of GM, then people may be more willing to accept any perceived risks [25,26], suggesting that it is important to understand how such benefits are perceived. Modifications perceived to primarily provide an economic advantage for the developer, such as faster growth rates, have been met with more negative public perceptions [27] than those regarded to have more widespread societal advantages, including improved nutrition [28], reduced use of chemicals [29], and reduced need for animals to undergo painful procedures [30]. These results suggest that the perceived benefits associated with the application of GM affect public support.

However, little experimental work has explored how the purpose behind GM of livestock affects public acceptance. Our primary aim was to investigate whether public attitudes toward applications of GM in dairy cattle differed when the purpose behind the GM was to improve animal welfare, increase worker safety or improve economic outcomes. To provide some generality to our findings, and to ground our work in real-world examples, we assessed the effect of the described purpose using two recently developed modifications in cattle: hornlessness and disease resistance.

## Materials and methods

The University of British Columbia Behavioral Research Ethics Board approved this study (protocol H17-01354).

### 2.1. Survey sample and design

One thousand and seventy-seven participants were recruited to take the survey using Amazon Mechanical Turk (www.mturk.com). Amazon Mechanical Turk provides researchers with a sample of individuals that are more diverse than groups generated from standard Internet samples while maintaining comparable reliability [31–33].

Participants first consented to the study. Participation was anonymous and voluntary, meaning participants could quit the survey at any time. Participants were introduced to our survey as follows:

“In the first part of this survey we want to know what you think about different applications of genetic modification for agriculture.”

“Genetic modification is the process of using biotechnology to alter the genetic information (DNA) of an organism to produce a certain trait.”

“Please tell us what you think about the following statements”

Next, each participant was randomly assigned to one of 10 scenarios using a 2 x 5 study design. The first treatment (with 2 levels) varied the application of genetic modification: modifying cattle to be hornless (i.e. polled) or disease resistant. The second treatment (with 5 levels) varied the described purpose for the GM: improved animal welfare, reduced costs, increased worker safety, all three purposes, or no purpose. See Table 1 for an overview of the 10 scenarios. Using a 7-point Likert scale, participants responded to how good (1 = *a very bad* thing, 7 = *a very good thing*), appropriate (1 = *totally appropriate*, 7 = *totally inappropriate*) and acceptable (1 = *completely unacceptable*, 7 = *completely acceptable*) they considered the proposed modification (n.b. reversal of the second question was used as an attention check for careful reading of responses; responses were recoded for analysis). The wording of these three questions was intentionally complementary and designed to allow the development of a construct used to assess attitudes to the scenario. Next, participants were prompted to “Feel free to say more about why you feel the way you do about this application of genetic modification” in an open-ended response.

**Table 1:** Overview of the 10 scenarios, assessing two applications of genetic modification (GM) and five different purposes for doing so.

Application	Purpose	Question wording for each scenario
Hornlessness	Animal welfare	“Genetically modifying cattle to be hornless in order to improve their welfare by eliminating the need for dehorning surgery would be…”
	Cost	“Genetically modifying cattle to be hornless in order to save costs by eliminating the need for dehorning surgery would be…”
	Worker safety	“Genetically modifying cattle to be hornless in order to protect farm workers who work with livestock from the risk of injury would be…”
	None	“Genetically modifying cattle to be hornless would be…”
	All	“Genetically modifying cattle to be hornless in order to improve animal welfare, save costs, and protect farm workers by eliminating the need for dehorning surgery would be…”
Disease resistance	Animal welfare	“Genetically modifying cattle to be disease resistant in order to improve their welfare would be…”
	Cost	“Genetically modifying cattle to be disease resistant in order to reduce the costs of veterinary treatment would be…”
	Worker safety	“Genetically modifying cattle to be disease resistant in order to reduce the risk of farm workers contracting a disease would be…”
	None	“Genetically modifying cattle to be disease resistant would be…”
	All	“Genetically modifying cattle to be disease resistant in order to improve animal welfare, eliminate the risk of workers contracting a disease, and reduce the costs associated with sick cows would be…”

We then assessed how effective the GM was perceived to be at achieving different purposes. Participants assigned to one of the ‘hornlessness’ scenarios were asked each of the following three questions: “How likely is it that genetically modifying cattle to be hornless will improve their welfare / help farmers save costs on veterinary treatment / will help protect workers?” Participants assigned to one of the ‘disease resistance’ scenarios were asked: “How likely is it that genetically modifying cattle to be disease resistant will improve their welfare / help farmers save costs on veterinary treatment / will help protect workers?” All questions were asked using a 7-point Likert scale with 1 = *very unlikely*, 7 = *very likely*.

Participants then responded to five true or false questions designed to test knowledge of GM [34]. Each of these five questions was accompanied by a follow-up question asking how certain they were about their response (1 = *not at all certain*, 5 = *very certain*). The order of these questions was randomized. Participants were then asked to rate their perceptions (*1 = strongly agree*, *5 = strongly disagree*) of 10 different types of animal use (e.g. for laboratory testing, breeding for skin, hunting), as part of the Attitudes toward Animal Scale [35]. Participants were also asked the same questions but from the perspective of the “average” American (“*Please tell us how much you think the average American agrees or disagrees with the following statements*”). Lastly, participants were asked a series of generic demographic questions including age, sex, income, education, marital status, number of children in household, political and religious affiliations, and a few project-specific demographic questions such as dietary preferences and number of pets. Upon completion, participants were debriefed, thanked, and paid 0.57 USD.

### 2.2. Sampling weights

Iterative proportional fitting was used to adjust the obtained sample to U.S. census data. Specifically, sex, age, and education microdata were obtained from the American Community Survey [36] and sample weights were generated accordingly using the *ipfweight* statement in Stata IC15.0 (StataCorp LP, College Station, TX). Weights were trimmed at a lower threshold of 0.2 and an upper threshold of 5 to avoid extreme influence of a particular data point [37]. All subsequent results are reported using these adjusted sample weights.

### 2.3. Analysis of quantitative survey data

Stata IC15.0 (StataCorp LP, College Station, TX) was used for statistical analysis, with a P-value < 0.05 considered significant. After ensuring internal consistency (Cronbach’s alpha = 0.90) an attitude score was created by averaging each participant’s answers to the questions of how good, appropriate, and acceptable they considered the GM. Knowledge of GM was calculated by adding up correct answers to the 5 knowledge questions, and confidence in knowledge was assessed by averaging the 5 confidence questions following the knowledge assessments (Cronbach’s alpha = 0.72). Additionally, two constructs were created for the self-perceived Attitudes toward Animals Scale (Cronbach’s alpha = 0.83) and perceived Attitudes toward Animals Scale for the average American (Cronbach’s alpha = 0.76).

To validate the efficacy of our purpose treatment we assessed whether the perceived effectiveness of GM to improve welfare, worker safety, or reduce cost was influenced by the initially provided purpose. A new variable was coded as ‘concordant’ if the question assessing perceived effectiveness was consistent with the purpose described in the scenario. For example, participants assigned to the animal welfare purpose group were coded as ‘concordant’ when asked about the perceived effectiveness of improving animal welfare. The other two questions concerning perceived effectiveness were coded as ‘discordant’. Participants in the group where no purpose was provided were always coded as ‘discordant,’ and participants where all three purposes were provided were always coded as concordant. We then assessed whether perceived effectiveness to achieve the purpose differed between concordant and discordant pairs using an univariable regression model.

The primary research aim was to assess attitudes toward GM depending on purpose (i.e. to improve animal welfare, reduce cost, and increase worker safety) separately for the disease-resistance and the hornless scenario using multivariable regression models adjusted for covariates. Use of separate models was supported by the absence of interaction effects between purpose and scenario. Additionally, two separate regression models per scenario were used because participants’ perceived effectiveness was influenced by the purpose provided as assessed previously. Therefore, to avoid perceived effectiveness biasing attitudes toward different purposes, we did not include this covariate in the model that was used to obtain regression coefficients for different purposes. Demographics, knowledge, confidence in knowledge, and scores of the Attitudes toward Animals Scale were included as covariates but these coefficients were not interpreted. Instead, a separate multivariable model was used to obtain regression coefficients for participants’ perceived effectiveness and all previously mentioned covariates. Although this model also included purpose, regression coefficients for this variable were deemed biased by effectiveness and ignored. Comparison of distinct groups that were not included in the multivariable regression model (e.g. participants’ attitudes between scenarios) were done using univariable analyses. To facilitate interpretation, belief in effectiveness was dichotomized based on the participant’s belief that it was at least ‘somewhat likely’ (score 5–7) that the GM was effective at achieving the specific purpose. Similarly, confidence in knowledge was dichotomized based on the median of 4 for regression analysis.

For each of the multivariable regression models, interaction terms that were plausible and statistically significant (P < 0.05) were considered. Backward elimination was based on assessment of Wald tests (variables were excluded if P < 0.05) and assessment of remaining variables for confounding (a change of the coefficients >15% was interpreted as confounding through the eliminated variable; in this case the variable was inserted back into the model).

### 2.4. Analysis of qualitative survey data

Representational thematic text analysis was applied to the open-ended responses [38]. Themes were identified through a combination of *a priori* [39] and emergent methods [40]; a coding dictionary from a companion study [30] was utilized and refined as responses from the current study were read. Two individuals trained in qualitative research each independently read a randomly selected sample of 80 open-ended responses, with 8 responses from each treatment group. These individuals independently identified themes that were present and then came together to discuss their findings. In this way, a document detailing the themes was established. Next, two individuals (one of whom was involved in the initial identification of themes) read and coded all remaining open-ended responses. These researchers individually coded 50 responses and then discussed discrepancies and agreed upon a final outcome. This method was repeated until all open-ended responses were coded. There were no limitations to the number of themes that could be present in one response and many responses contained multiple themes. In the following sections, quotes from the open-ended responses written by survey participants will be provided alongside an identifying, but anonymous label consisting of a respondent ID (e.g. R10) and an abbreviation for the scenario they were provided with (e.g. D_AW). Abbreviations are as follows: “D” symbolizes a ‘disease resistance’ application, “H” a ‘hornlessness’ application. “AW” means that the purpose provided in the treatment was animal welfare, “CO” was cost, “WS” was worker safety, “ALL” was all three, and “NONE” was no purpose given (see Table 1 for all scenarios).

To create a word cloud, words that were mentioned fewer than 4 times as well as words that did not provide meaningful content (e.g., also, therefore, animal) were deleted. This was done to filter for the most relevant words and enhance readability of the final word cloud. Then, responses were copied into an online software tool (wordclouds.com) to graphically display the words used more frequently in larger fonts.

We also assessed whether participants’ mentioning of a specific theme was associated with their attitude score toward GM using linear regression analyses with the binary predictor variables (mentioning of theme) adjusted for the covariates age, education, sex, and treatment.

## Results and discussion

We excluded respondents from the analysis who were < 19 years old (n = 3), did not finish the survey (n = 67), or did not vary in their answers to the questions related to the ethical use of animals (n = 50; these invariant responses were taken as evidence of insufficient care in answering). After these exclusions, the total sample for the quantitative analysis was 957 participants. On average, answers to the qualitative question about participants’ attitudes were 23 words long with 81 participants not providing an answer. Responses (n = 167) that provided no useful content or were deemed to have unclear meanings were excluded, leaving 709 open-ended responses.

### 3.1. Scenario and attitude toward GM

**Fig 1:**
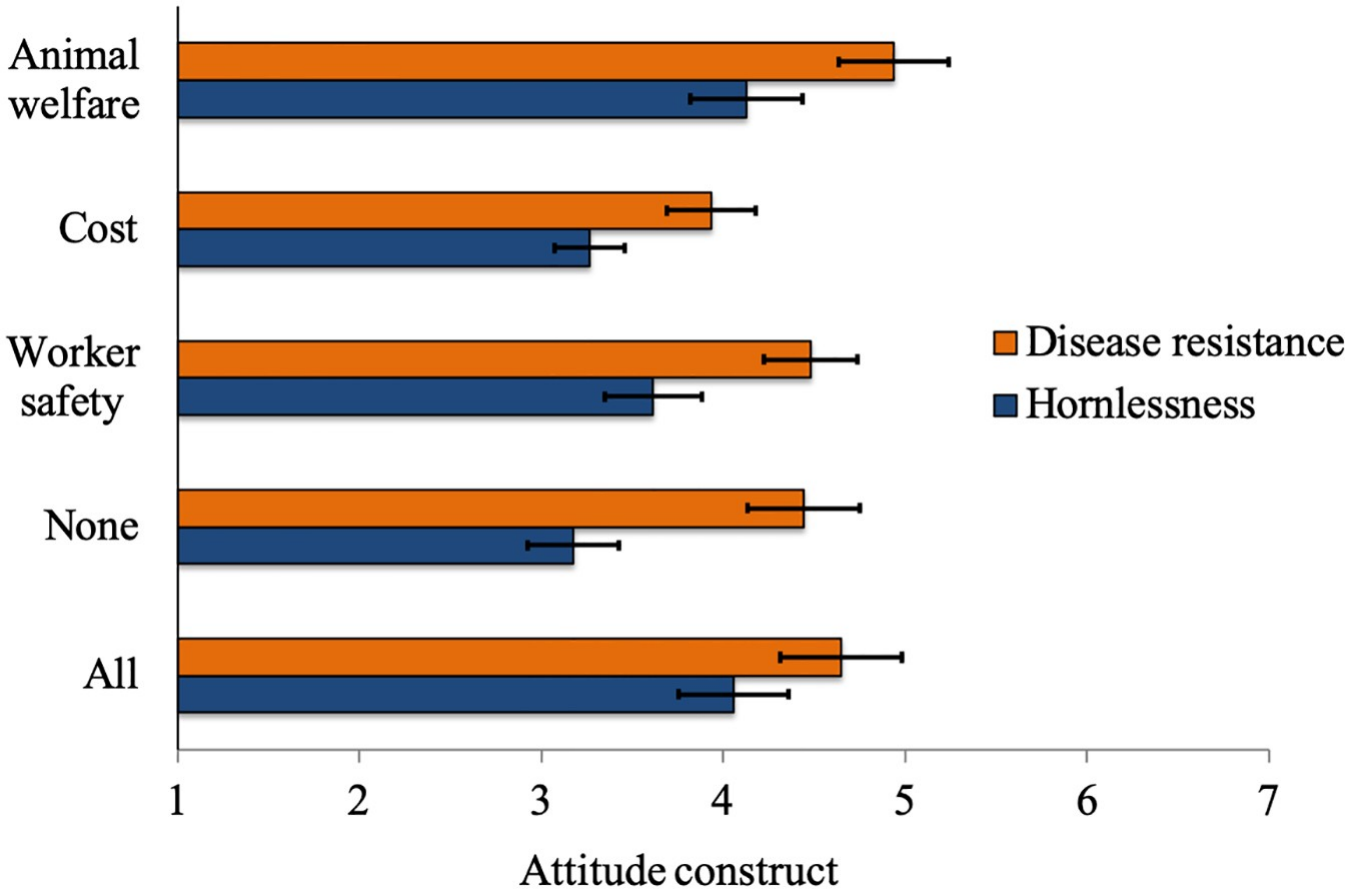
Attitude construct (1 = *most negative attitude score*; 7 = *most positive attitude score*)^1^ of 957 survey participants toward genetic modification (GM) in dairy cattle. Responses were stratified by application of the GM (i.e. disease resistant or hornless cattle) and by purposes of the GM (i.e. improving animal welfare, reducing cost for the farmer, increasing worker safety, all purposes, or no purpose provided). Least-square means and standard errors are adjusted for covariates based on multivariable linear regression.^1^Three questions assessing participant attitudes toward GM on 7-point Likert scales were used to create an attitude construct.

Overall, based on scales ranging from 1 to 7, the attitude score averaged 4.0 (SE ± 0.10); hence participants appeared to be ambivalent toward GM in dairy cattle. Participants assigned to the ‘disease resistance’ application were more supportive than participants in the ‘hornlessness’ application (4.5 ± 0.15 vs. 3.7 ± 0.14; P < 0.001). For the ‘disease resistance’ group, attitudes toward GM were lowest when the purpose was reducing costs, and were highest for animal welfare; this difference was statistically significant (contrast = 1.00; 95% CI = 0.12–1.88; P = 0.03; Fig 1). In the ‘hornlessness’ group attitudes were highest when the purpose was animal welfare or when all three purposes were provided (Fig 1). Specifically, attitudes were significantly higher when the purpose of animal welfare was contrasted against the purpose of cost reduction (contrast = 0.86; 95% CI = 0.09–1.64; P = 0.03) or compared to no purpose (contrast = 0.95; 95% CI = 0.26–1.64; P = 0.01). Additionally, when all purposes were provided, participants had higher attitudes scores compared to the purpose of cost reduction (contrast = 0.79; 95% CI = 0.02–1.56; P = 0.04) and when no purpose was provided (contrast = 0.88; 95% CI = 0.19–1.58; P = 0.01). It appears that participants in the disease-resistance group were able to envisage the benefits of disease resistance even when no purpose was provided, but few people were aware of the practice of dehorning or recognized the potential issues associated with it [41].

**Fig 2:**
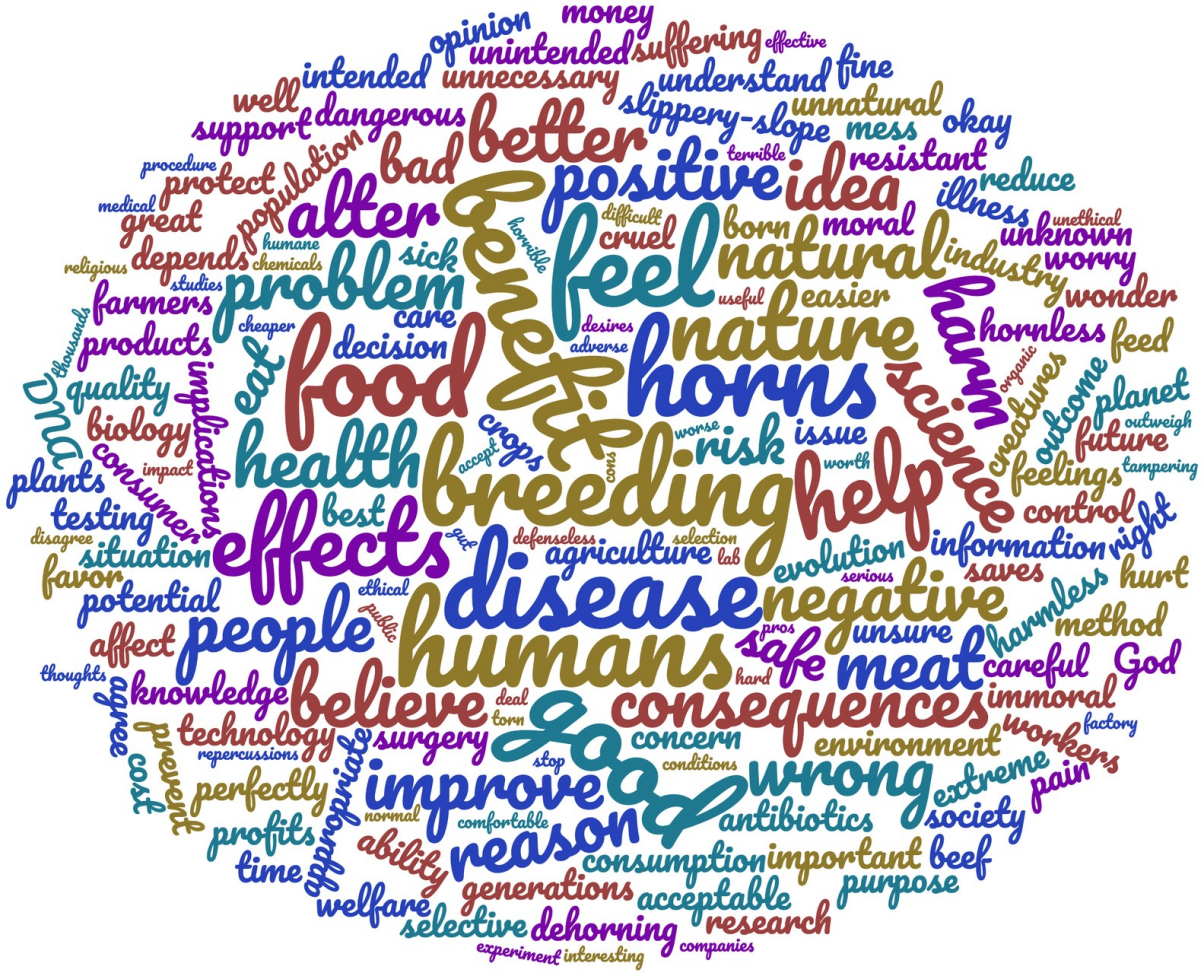
Word cloud of participant responses to the open-ended prompt: “Feel free to say more about why you feel the way you do about this application of genetic modification”. Participants were provided with one of two different applications of GM (i.e. disease resistant or hornless cattle) and one of five different purposes (i.e. improving animal welfare, reducing cost for the farmer, increasing worker safety, all purposes, or no purpose provided). Word cloud includes words that provided meaningful content and were mentioned at least 4 times. Larger words represent more frequent answers.

Qualitative responses (see Fig 2 and Table 2) often reflected discontent with GM, independent of the scenario provided. Responses coded as ‘opposition to GM’ often consisted of a short statement denouncing GM (e.g. “Genetic modification is wrong on all counts. It should never be done” R88, H_CO and “I don’t approve of genetic modification, period” R447, D_WS). Consequently, participants who brought up opposition to GM in their qualitative answers had more negative attitudes toward GM compared to those that did not mention this theme (Table 2). Some respondents elaborated on the reasons for their opposition by referring to a specific purpose of GM. For example, many respondents expressed opposition to the practice of dehorning as well as to genetically modifying cows to be hornless (e.g. “I’m unsure why we need to dehorn them. It seems like a flimsy motivation for genetically altering cattle” R73, H_AW). In the disease resistance scenarios, disapproval was expressed toward genetic modification being used as a Band-Aid solution (e.g. “If cattle are kept in healthy conditions, there would be no need for genetic modification for disease resistance. If they are carefully bred, raised, and taken care of, diseases would be infrequent.” R625, D_NONE).

**Table 2:** Themes identified from 709 responses to open-ended questions and their relationship to participant attitudes toward genetic modification (GM) in dairy cattle^1^.

Theme	Key elements	Disease resistance (%)	Hornlessness (%)	Coefficient (95% Confidence Interval); p-value^2^
Animal welfare	Cattle well-being, pain, quality of life, health, or affect	27.8	45.0	0.65 (0.25–1.10) P = 0.002
Naturalness	Naturalness, natural processes, or nature and the environment	19.0	24.3	-0.99 (-1.62 –(-0.37)) P = 0.002
Morality	Perception of right and wrong, moral framework, personal beliefs, religion	20.8	20.6	-0.89 (-1.35 –(-0.42)) P<0.001
Uncertainty	Unintended side effects or consequences, desire for more testing, fear of unknown	25.1	14.6	-1.14 (-1.62 –(-0.66)) P<0.001
Oppose GM	An explicitly stated negative attitude toward GM	17.8	19.0	-2.18 (-2.57 –(-1.80)) P<0.001
Consumption	Food product quality, taste, yield, availability, appearance, nutritional value, or safety or consumer health, safety, or satisfaction	21.1	9.8	0.43 (-0.14–0.99) P = 0.14
Oppose treatment	A dissatisfaction with a specific purpose or outcome of GM	9.7	16.7	-1.35 (-2.19 –(-0.51)) P = 0.002
Worker welfare	Farm worker safety, health, happiness, and general well-being	8.8	7.9	1.37 (0.94–1.80) P<0.001
Economics	Financial considerations for farmers, consumers, scientists, or companies	11.2	5.6	0.73 (0.15–1.30) P = 0.01

^1^Themes were not exclusive; participant reasons often included multiple themes, and different participants sometimes used the same themes to both support and oppose GM. Key elements included in the theme, and the % of responses in which the theme was referenced are reported for participants randomly assigned to read about GM in cattle to either improve disease resistance or produce hornlessness in cattle. Within each of these two applications, participants were assigned to different descriptions of the purported purpose of GM (i.e. improving animal welfare, reducing cost for the farmer, increasing worker safety, all purposes, or no purpose provided).

^2^Linear regression analysis between whether or not participants mentioned the particular theme (binary predictor) and their attitude score with treatment, sex, age and education as covariates.

Animal welfare was the most common theme in the qualitative analysis (37% of responses) and results from this survey are consistent with earlier work indicating that citizens generally care about dairy cattle welfare [42]. Many respondents expressed the hope that modifications would be helpful (e.g. “I would like the cows to be healthy and happy” R680, D_ALL and “[genetic modification] saves the cow the surgery” R143, H_WS) and bringing up the theme of animal welfare was positively associated with participant attitudes toward GM (Table 2). Nevertheless, others worried about negative effects of GM on well-being (e.g. “If genetic modification does not harm animals in any way, then I am probably okay with it” R470, D_WS). In the hornless treatments, some respondents were worried that horns serve an important purpose to cattle (e.g. “…modifying a cow’s horns could be detrimental to the survival of the cow. It may need [horns] for protection.” R179, H_WS).

Compared to animal welfare, worker safety and economic considerations were less frequently mentioned in the responses to the open-ended questions (8.3% and 8.2%, respectively). However, participants suggested that proposed modifications could be beneficial for workers (e.g. “Horns can be dangerous to humans and other animals, so we need to pick the least painful way to remove them” R25, H_AW, and “It helps reduce the risk to farm workers and also to the cattle themselves” R449, D_WS). Consequently, mentioning of worker safety was positively related to attitudes toward GM (Table 2). The qualitative theme “economics” included responses discussing the impact on farmers, consumers, and other groups. In general, genetic modification was deemed to be economically beneficial for farmers and consumers and mentioning of this theme was positively associated with attitudes toward GM (e.g. “I see this as a win-win situation for farmers and the general population. Farmers would be able to raise better cattle for meat and have to spend less on medicine and veterinary bills.” R528, D_CO and “Anything to reduce costs is a good thing, and it’d make beef cheaper over time” R533, D_CO). However, some expressed discontent with corporations and other organizations benefiting financially (e.g. “I don’t support GMOs because I think most of the companies that sell them are evil and just want to push out profits. I do support GMOs by scientists if they are doing it safely and not with malicious intent” R403, D_AW). This statement highlights the importance of trust between the public and GM developers. The distrust that many citizens have in corporations [43] may pose an important barrier to adoption.

### 3.2. Other factors associated with participant attitudes toward GM

#### 3.2.1 Perceived effectiveness of GM

Participants rated the effectiveness of the GM higher if they were assigned to a concordant (mean ± SE: 4.7 ± 0.10) versus discordant treatment (4.4 ± 0.07; P < 0.01), illustrating that the provided purpose influenced perceived effectiveness.

**Fig 3:**
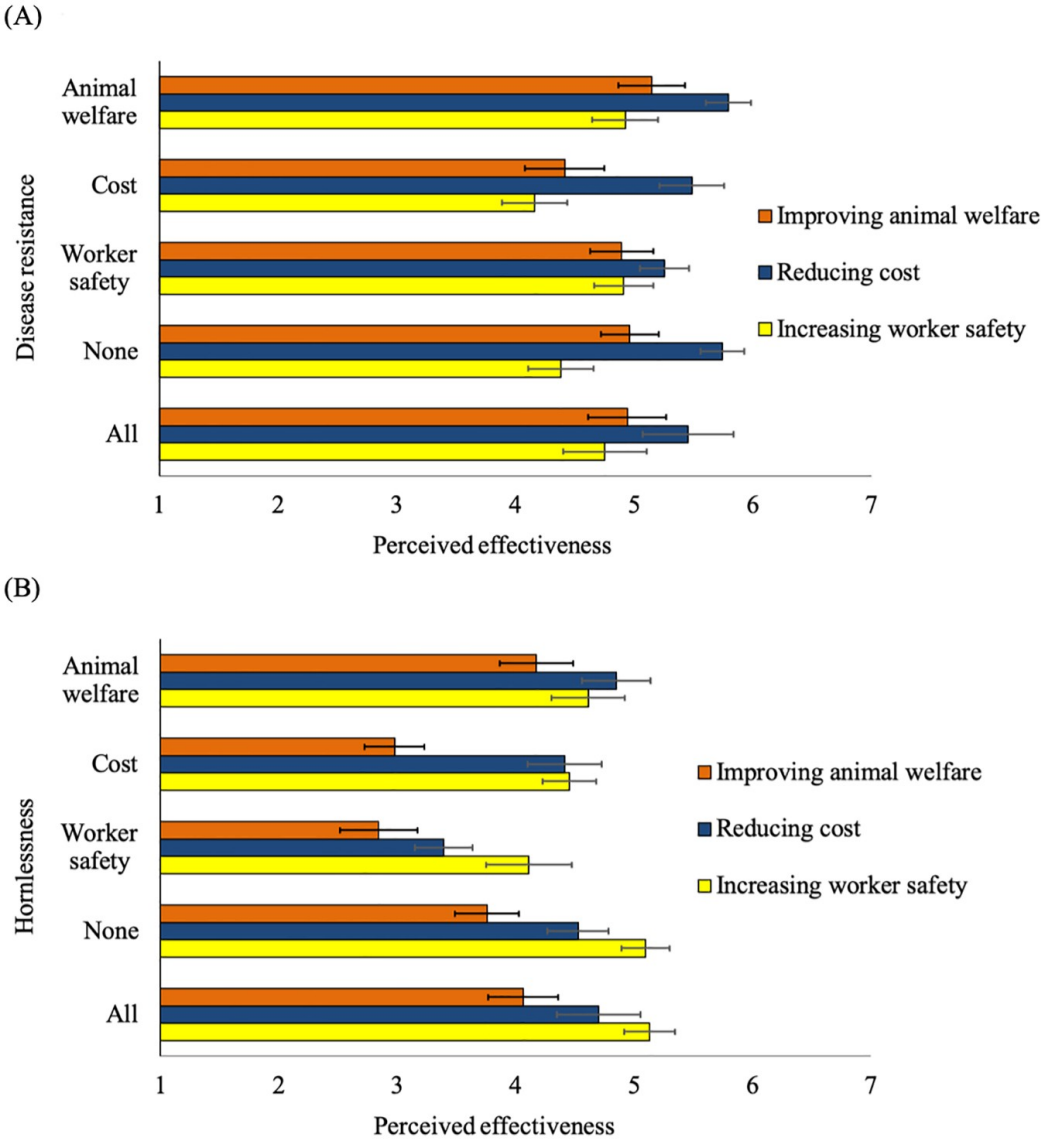
Survey participants’ (n = 957) belief in the effectiveness of genetic modification (GM) in dairy cattle stratified by application of the GM (i.e. disease resistant (A) or hornless cattle (B)). Responses within the applications were further stratified by purpose of the GM (i.e. improving animal welfare, reducing cost for the farmer, increasing worker safety, all purposes, or no purpose provided). Responses were rated on 7-point Likert scales (1 = *very unlikely*, 7 = *very likely*) whether participants believed GM could improve animal welfare, reduce cost for the farmer or increase worker safety. Unadjusted means and standard errors are displayed.

Overall, participants in the ‘disease resistance’ group assigned a mean score of 5.0 ± 0.12 to the effectiveness of GM to achieve any purpose, which was higher than that of the ‘hornlessness’ group (4.2 ± 0.12; P < 0.001). In the ‘disease resistance’ group, the perceived effectiveness in reducing cost had the highest value across purpose groups (Fig 3A), and in the ‘hornlessness’ group, belief in the effectiveness in improving animal welfare was, overall, lowest (Fig 3B). Perhaps many participants were not aware that dehorning is painful and is mostly done without pain medication, and thus failed to see a connection between eliminating the need for dehorning and improving animal welfare. Earlier studies that specified this connection more clearly reported negative reactions, with some participants describing dehorning without pain relief as cruel or inhumane [30,44].

Perceived effectiveness is an important determinant of peoples’ attitudes, for example, when considering participation in a rally [45] or a recycling program [46]. Similarly, attitudes toward GM were largely associated with their perception that the GM is successful in achieving a specific purpose. The perceived effectiveness in improving animal welfare had the highest association with attitudes in the ‘disease resistance’ group (Coefficient = 1.83; 95% CI = 1.34–2.32; P < 0.001) and the ‘hornlessness’ group (Coefficient = 1.25; 95% CI = 0.78–1.72; P < 0.001). Although the relationship between perceived effectiveness in increasing worker safety and attitude was also significant, coefficients were substantially lower (‘disease resistance’ group: Coefficient = 0.70; 95% CI = 0.20–1.19; P = 0.01, ‘hornlessness’ group: Coefficient = 1.00; 95% CI = 0.47–1.55; P < 0.001). Furthermore, perceived effectiveness in reducing costs had the lowest coefficient of all ‘effectiveness’ measures in the ‘disease-resistance’ group (Coefficient = 0.62; 95% CI = 0.13–1.11; P = 0.01) and in the ‘hornlessness’ group (Coefficient = 0.59; 95% CI = 0.08–1.09; P = 0.02). We conclude that attitudes toward GM are positively influenced by belief in effectiveness, especially regarding public goods like animal welfare and worker safety.

#### 3.2.2. Demographics

Use of sample weights adjusted the study sample demographics to be more similar to the US population. Like the US census data [36], the sample included 52% females. Mean age of respondents was 47 years (mean age US population >18 years is 50 years [36]. Education in the sample population was higher than in the U.S. census data (50% had a bachelor’s degree or higher compared to 31% of the U.S. population >18 years), Similarly, with 44% of participants identifying themselves as liberal, this group was overrepresented in the study even after adjustment using sample weights [47]. However, lack of a significant association with attitudes toward GM suggests these deviations do not jeopardize external validity of the study results.

Males had more positive attitudes toward GM, and this difference was significant in the ‘disease-resistance’ group (Coefficient = 0.36; 95% CI = 0.01–0.72; P = 0.04). In the ‘hornlessness’ group, vegetarians or vegan participants had more negative attitudes toward GM (Coefficient = -0.46; 95% CI = -0.80- (-0.11); P = 0.01). Previous studies (reviewed by [48]) have often described associations between attitudes toward GM and demographic factors such as gender (e.g. females tend to be more negative) and country of residence (e.g. European consumers tend to be more critical). In this study, factors such as the provided purpose or scenario, as well as perceived effectiveness in achieving the purpose, appeared to be more relevant than demographic factors. Because results are often divergent in terms of demographics, Costa Font et al. [48] suggested that individual values and their interaction with knowledge should be considered as key determinants underpinning attitudes.

#### 3.2.3. Knowledge and confidence in knowledge

Of the 5 knowledge questions, participants answered a mean of 3.9 (SE ± 0.5) correctly. Confidence in this knowledge was, on average, 3.7 on a scale of 1–5 (SE ± 0.5). Approximately 21% of participants who rated their confidence as 5 out of 5 were extremely negative toward GM (ranking of 1 on a scale of 7), compared to 10–14% for participants with lower confidence levels. This result is consistent with findings of Fernbach et al. [49] showing that opponents of GM often consider themselves as knowledgeable.

Participant attitudes were positively related with knowledge in the ‘disease-resistance’ group (Coefficient = 0.22; 95% CI = 0.06–0.38; P = 0.01). Similarly, McConnachie et al. [30] reported that knowledge was positively associated with attitudes and willingness to consume products from GM animals. Perhaps a lack of knowledge about GM is associated with a more cautious outlook toward GM animals [50]. This feeling of uncertainty toward the outcomes or consequences of GM was expressed in approximately 20% of qualitative responses and participants who mentioned uncertainty had more negative attitudes toward GM (Table 2). For example, one participant stated “We’re tinkering with things which may have dire unintended consequences and it’s difficult to properly test the outcomes” (R19, H_AW), and another reported “I am concerned about the long-term risks” (R464, D_WS). This uncertainty was highlighted by some participants especially regarding consumption of food products (e.g. “we don’t know how GMOs will affect beef or dairy.” R62, H_AW). A desire for food labelling was prevalent in the “consumption’ theme as well (e.g. “I believe that as of today, GMO foods should be labelled but [I] am open to GMO foods that have been safely tested.” R437, D_AW).

#### 3.2.4. Attitudes toward animals

The Attitudes toward Animal Scale has been used in a variety of studies (e.g. [51,52]) and has excellent concurrent and convergent validity [35]. The brief version of this scale used for this study consisted of 10 items but is highly correlated with the 20-item scale [35]. Self-perceived attitudes toward animals were more positive than those perceived for the average American (3.4 ± 0.04 vs. 2.9 ± 0.03; P < 0.001). Self-perceived attitudes toward animals were negatively associated with attitudes toward GM in multivariable regression analyses in the ‘hornlessness’ group (Coefficient = -0.31; 95% CI = -0.62-(-0.004); P = 0.047). However, perceived attitudes toward animals for the average American showed no relationship with attitude toward GM. This divergence could either be explained by participants’ biased responses when asked about their own perceptions (i.e. a social desirability bias [53]) or by a belief that their perceptions were truly different from those of the average American. The questions that comprised the Attitudes toward Animals Scale largely dealt with the ethical use of animals by humans, which was also one of the nine themes identified in the qualitative analysis (Table 2). Some respondents regarded it as moral responsibility to improve animal welfare in any possible way (e.g. “what improvements we can make though science should be made” R438, D_AW), but others argued that “it’s not our job to change [animals] to suit our needs” R185, H_WS. Similarly, naturalness is important for many citizens, not only when discussing GM foods [54] but also when considering health products [55] and farm animal husbandry [56]. In this study, many respondents noted that GM was unnatural (e.g. “it feels completely unnatural” R72, H_AW and “goes against nature” R552, D_CO). Consequently, participants who mentioned the themes ‘morality’ or ‘naturalness’ in their qualitative responses were more negative in their attitudes toward GM (Table 2).

## Conclusions and implications

This study contributes understanding of public attitudes toward GM in farm animals, especially as affected by different perceived purposes including improved animal welfare. The results illustrate that attitudes depend upon the specifics of the scenario; in this case attitudes were more positive when the modification induced disease resistance rather than hornlessness, perhaps because participants found it easier to imagine the impact of disease resistance whereas hornlessness was likely unfamiliar. Perceived effectiveness appeared to be a key factor influencing attitudes toward GM; acceptance of GM technology would likely benefit from evidence that it is successful at achieving intended purposes. These results suggest citizens are more likely to accept GM technologies when these provide demonstrated public goods, including for the affected animals.

## Supporting information

S1 File Ritter et al survey. Online survey instrument used to assess US citizens’ attitudes toward genetic modification in dairy cattle.
(PDF)

S2 File Ritter et al dataset. Data describing US citizens attitudes toward genetic modification in dairy cattle.
(XLSX)
