# Supplementary material for: Public attitudes toward genetic modification in dairy cattle
Source: PLoS One. 2019 Dec 2;14(12):e0225372. doi: 10.1371/journal.pone.0225372 (PMC6886766; doi:10.1371/journal.pone.0225372)
Supplement: S1 File — Online survey instrument used to assess US citizens’ attitudes toward genetic modification in dairy cattle. (PDF) [file pone.0225372.s001.pdf]

Genetic Modification and Animal Welfare - Adam

Default Question Block Block Options

Q1

Consent

Principal Investigator: Dr. Marina von Keyserlingk, Professor and NSERC Industrial Research Chair, University of British Columbia, marina.vonkeyserlingk@ubc.ca 604.822.4898

Co-Investigator: Jesse Robbins, Post-Doctoral Fellow, University of British Columbia, 604.822.5715

Funding
This research is funded by the University of British Columbia.

Recruitment
Participation is this project is open to anyone over 18 years of age. You were recruited to participate in this study using Amazon's Mechanical Turk service.

Risks
There are no known risks in taking this survey. You may leave at any point during the survey.

Confidentiality
We will not collect any information that allows us to identify or contact you individually. The data you provide will be stored in a secure database and will only be accessible to the research team. This online survey is hosted by Qualtrics, a web survey company located in the USA. If you choose to participate, in the survey you understand that your responses will be stored in the USA. The privacy and security policy of the survey service provider can be found at: http://www.qualtrics.com/privacy-statement/.

If you have any concerns or complaints about your rights as a research participant and/or your experiences while participating in this study, contact the Research Participant Complaint Line in the UBC Office of Research Ethics at 604-822-8598 or if long distance e-mail RSIL@ors.ubc.ca or call toll free 1-877-822-8598.

Consent
I am over 18 years of age and I understand my participation in this study is entirely voluntary and that I may choose to quit at anytime. I understand that any information I provide will be anonymous and will only be stored and analyzed for the purposes of this research.

YES, I consent

Part 1: Introduction Block Options

Q3

In the first part of this survey we want to know what you think about different applications of genetic modification for agriculture.

Genetic modification is the process of using biotechnology to alter the genetic information (DNA) of an organism to produce a certain trait.

Please tell us what you think about the following statements:

Welf1 Randomized Block Options

W1a

Genetically modifying cattle to be hornless in order to improve their welfare by eliminating the need for dehorning surgery would be...

a very bad thing			neither good nor bad			a very good thing
○	○	○	○	○	○	○

W1b

Genetically modifying cattle to be hornless in order to improve their welfare by eliminating the need for dehorning surgery would be...

totally appropriate			neither appropriate nor inappropriate			totally inappropriate
○	○	○	○	○	○	○

W1c

Genetically modifying cattle to be hornless in order to improve their welfare by eliminating the need for dehorning surgery would be...

completely unacceptable			neither acceptable nor unacceptable			completely acceptable
○	○	○	○	○	○	○

W1d

Feel free to say more about why you feel the way you do about this application of genetic modification

Welf2 Randomized

Block Options

W2a

Genetically modifying cattle to be hornless in order to save costs by eliminating the need for dehorning surgery would be...

a very bad thing			neither good nor bad			a very good thing
○	○	○	○	○	○	○

W2b

Genetically modifying cattle to be hornless in order to save costs by eliminating the need for dehorning surgery would be...

totally appropriate			neither appropriate nor inappropriate			totally inappropriate
○	○	○	○	○	○	○

W2c

Genetically modifying cattle to be hornless in order to save costs by eliminating the need for dehorning surgery would be...

completely unacceptable			neither acceptable nor unacceptable			completely acceptable
○	○	○	○	○	○	○

W2d

Feel free to say more about why you feel the way you do about this application of genetic modification

Welf3 Randomized

Block Options

W3a

Genetically modifying cattle to be hornless in order to protect farm workers who work with livestock from the risk of injury would be...

a very bad thing			neither good nor bad			a very good thing
○	○	○	○	○	○	○

W3b

Genetically modifying cattle to be hornless in order to protect farm workers who work with livestock from the risk of injury would be...

totally appropriate			neither appropriate nor inappropriate			totally inappropriate
○	○	○	○	○	○	○

W3c

Genetically modifying cattle to be hornless in order to protect farm workers who work with livestock from the risk of injury would be...

completely unacceptable			neither acceptable nor unacceptable			completely acceptable
○	○	○	○	○	○	○

W3d

Feel free to say more about why you feel the way you do about this application of genetic modification

Welf4 Randomized

Block Options

W4a

Genetically modifying cattle to be hornless would be...

a very bad thing			neither good nor bad			a very good thing
○	○	○	○	○	○	○

W4b

Genetically modifying cattle to be hornless would be...

totally appropriate			neither appropriate nor inappropriate			totally inappropriate
○	○	○	○	○	○	○

W4c

Genetically modifying cattle to be hornless would be...

completely unacceptable			neither acceptable nor unacceptable			completely acceptable
○	○	○	○	○	○	○

W4d

Feel free to say more about why you feel the way you do about this application of genetic modification

Welf5 Randomized

Block Options

W5a

Genetically modifying cattle to be hornless in order to improve animal welfare, save costs, and protect farm workers by eliminating the need for dehorning surgery would be...

a very bad thing			neither good nor bad			a very good thing
○	○	○	○	○	○	○

W5b

Genetically modifying cattle to be hornless in order to improve animal welfare, save costs, and protect farm workers by eliminating the need for dehorning surgery would be...

totally appropriate			neither appropriate nor inappropriate			totally inappropriate
○	○	○	○	○	○	○

W5c

Genetically modifying cattle to be hornless in order to improve animal welfare, save costs, and protect farm workers by eliminating the need for dehorning surgery would be...

completely unacceptable			neither acceptable nor unacceptable			completely acceptable
○	○	○	○	○	○	○

W5d

Feel free to say more about why you feel the way you do about this application of genetic modification

Dis1 Randomized — Block Options

D1a

Genetically modifying cattle to be disease-resistant in order to improve their welfare would be...

a very bad thing			neither good nor bad			a very good thing
○	○	○	○	○	○	○

D1b

Genetically modifying cattle to be disease-resistant in order to improve their welfare would be...

totally appropriate			neither appropriate nor inappropriate			totally inappropriate
○	○	○	○	○	○	○

D1c

Genetically modifying cattle to be disease-resistant in order to improve their welfare would be...

completely unacceptable			neither acceptable nor unacceptable			completely acceptable
○	○	○	○	○	○	○

D1d

Feel free to say more about why you feel the way you do about this application of genetic modification

Dis2 Randomized — Block Options

D2a

Genetically modifying cattle to be disease-resistant in order to reduce the risk of farm workers contracting a disease would be...

a very bad thing			neither good nor bad			a very good thing
○	○	○	○	○	○	○

D2b

Genetically modifying cattle to be disease-resistant in order to reduce the risk of farm workers contracting a disease would be...

totally appropriate			neither appropriate nor inappropriate			totally inappropriate
○	○	○	○	○	○	○

D2c

Genetically modifying cattle to be disease-resistant in order to reduce the risk of farm workers contracting a disease would be...

completely unacceptable			neither acceptable nor unacceptable			completely acceptable
○	○	○	○	○	○	○

D2d

Feel free to say more about why you feel the way you do about this application of genetic modification

Dis3 Randomized Block Options

D3a

Genetically modifying cattle to be disease-resistant in order to reduce the costs of veterinary treatment would be...

a very bad thing			neither good nor bad			a very good thing
○	○	○	○	○	○	○

D3b

Genetically modifying cattle to be disease-resistant in order to reduce the costs of veterinary treatment would be...

totally appropriate			neither appropriate nor inappropriate			totally inappropriate
○	○	○	○	○	○	○

D3c

Genetically modifying cattle to be disease-resistant in order to reduce the costs of veterinary treatment would be...

completely unacceptable			neither acceptable nor unacceptable			completely acceptable
○	○	○	○	○	○	○

D3d

Feel free to say more about why you feel the way you do about this application of genetic modification

Dis4 Randomized — Block Options

D4a

Genetically modifying cattle to be disease-resistant would be...

a very bad thing			neither good nor bad			a very good thing
○	○	○	○	○	○	○

D4b

Genetically modifying cattle to be disease-resistant would be...

totally appropriate			neither appropriate nor inappropriate			totally inappropriate
○	○	○	○	○	○	○

D4c

Genetically modifying cattle to be disease-resistant would be...

completely unacceptable			neither acceptable nor unacceptable			completely acceptable
○	○	○	○	○	○	○

D4d

Feel free to say more about why you feel the way you do about this application of genetic modification

Dis5 Randomized — Block Options

D5a

Genetically modifying cattle to be disease-resistant in order to improve animal welfare, eliminate the risk of workers contracting a disease, and reduce the costs associated with sick cows would be...

a very bad thing			neither good nor bad			a very good thing
○	○	○	○	○	○	○

D5b

Genetically modifying cattle to be disease-resistant in order to improve animal welfare, eliminate the risk of workers contracting a disease, and reduce the costs associated with sick cows would be...

totally appropriate			neither appropriate nor inappropriate			totally inappropriate
○	○	○	○	○	○	○

D5c

Genetically modifying cattle to be disease-resistant in order to improve animal welfare, eliminate the risk of workers contracting a disease, and reduce the costs associated with sick cows would be...

completely unacceptable			neither acceptable nor unacceptable			completely acceptable
○	○	○	○	○	○	○

D5d

Feel free to say more about why you feel the way you do about this application of genetic modification

Welf_Beliefs Randomized

Block Options

Q126

How likely is it that genetically modifying cattle to be hornless will improve their welfare?

Very unlikely	Moderately unlikely	Somewhat unlikely	Neither likely nor unlikely	Somewhat likely	Moderately likely	Very likely
○	○	○	○	○	○	○

Q127

How likely is it that genetically modifying cattle to be hornless will help protect workers?

Very unlikely	Moderately unlikely	Somewhat unlikely	Neither likely nor unlikely	Somewhat likely	Moderately likely	Very likely
○	○	○	○	○	○	○

Q128

How likely is it that genetically modifying cattle to be hornless will help farmers save costs on veterinary treatment?

Very unlikely	Moderately unlikely	Somewhat unlikely	Neither likely nor unlikely	Somewhat likely	Moderately likely	Very likely
○	○	○	○	○	○	○

Dis_Beliefs Randomized

Block Options

Q157

How likely is it that genetically modifying cattle to be disease-resistant will improve their welfare?

Very unlikely	Moderately unlikely	Somewhat unlikely	Neither likely nor unlikely	Somewhat likely	Moderately likely	Very likely
○	○	○	○	○	○	○

Q158

How likely is it that genetically modifying cattle to be disease-resistant will help protect workers?

Very unlikely	Moderately unlikely	Somewhat unlikely	Neither likely nor unlikely	Somewhat likely	Moderately likely	Very likely
○	○	○	○	○	○	○

Q159

How likely is it that genetically modifying cattle to be disease-resistant will help farmers save costs on veterinary treatment?

Very unlikely	Moderately unlikely	Somewhat unlikely	Neither likely nor unlikely	Somewhat likely	Moderately likely	Very likely
○	○	○	○	○	○	○

OBJ_Knowledge — Block Options

Q68

Some people tend to know very little about genetic modification, while others know quite a lot. In the next section we want to see how much you know about genetic modification.

Know1 — Block Options

K1

Genetically modified cows contain genes while ordinary cows do not.

- True
- False

Display This Question:

If Genetically modified cows contain genes while ordinary cows do not. True Is Selected

Or Genetically modified cows contain genes while ordinary cows do not. False Is Selected

CK1

How certain are you about your answer?

Not at all certain				Very certain
○	○	○	○	○

Know2 — Block Options

K2

Genetically modified animals are always bigger than ordinary animals

- True
- False

Display This Question:

If Genetically modified animals are always bigger than ordinary animals True Is Selected

Or Genetically modified animals are always bigger than ordinary animals False Is Selected

CK2

How certain are you about your answer?

Not at all certain				Very certain
○	○	○	○	○

Know3 Block Options

K3

Cows modified with genes from a catfish would probably taste fishy.

- True
- False

Display This Question:

If Cows modified with genes from a catfish would probably taste fishy. True Is Selected

Or Cows modified with genes from a catfish would probably taste fishy. False Is Selected

CK3

How certain are you about your answer?

Not at all certain				Very certain
○	○	○	○	○

Know4 Block Options

K4

By eating a genetically modified product, a person's genes could also become modified.

- True
- False

Display This Question:

If By eating a genetically modified product, a person's genes could also become modified. True Is Selected

Or By eating a genetically modified product, a person's genes could also become modified. False Is Selected

CK4

How certain are you about your answer?

Not at all certain				Very certain
○	○	○	○	○

Know5 Block Options

K5

It is possible to transfer plant genes into animals.

- True
- False

Display This Question:

If It is possible to transfer plant genes into animals. True Is Selected

Or It is possible to transfer plant genes into animals. False Is Selected

CK5

How certain are you about your answer?

Not at all certain				Very certain
○	○	○	○	○

Direct_brief Randomized Block Options

Q166

Now, please tell us how much ***YOU*** agree or disagree with the following statements.

Q167

It is morally wrong to hunt wild animals just for sport.

Strongly Agree	Agree	Neither Agree nor Disagree	Disagree	Strongly Disagree
○	○	○	○	○

Q168

I do not think that there is anything wrong with using animals in medical research.

Strongly Agree	Agree	Neither Agree nor Disagree	Disagree	Strongly Disagree
○	○	○	○	○

Q169

I think it is perfectly acceptable for cattle and hogs to be raised for human consumption.

Strongly Agree	Agree	Neither Agree nor Disagree	Disagree	Strongly Disagree
○	○	○	○	○

Q170

Basically, humans have the right to use animals as we see fit.

Strongly Agree	Agree	Neither Agree nor Disagree	Disagree	Strongly Disagree
○	○	○	○	○

Q171

The slaughter of whales and dolphins should be immediately stopped even if it means some people will be put out of work.

Strongly Agree	Agree	Neither Agree nor Disagree	Disagree	Strongly Disagree
○	○	○	○	○

Q172

I sometimes get upset when I see wild animals in cages at zoos.

Strongly Agree	Agree	Neither Agree nor Disagree	Disagree	Strongly Disagree
○	○	○	○	○

Q173

Breeding animals for their skins is a legitimate use of animals.

Strongly Agree	Agree	Neither Agree nor Disagree	Disagree	Strongly Disagree
○	○	○	○	○

Q174

Some aspects of biology can only be learned through dissecting preserved animals such as cats.

Strongly Agree	Agree	Neither Agree nor Disagree	Disagree	Strongly Disagree
○	○	○	○	○

Q175

It is unethical to breed purebred dogs for pets when millions of dogs are killed in animal shelters each year.

Strongly Agree	Agree	Neither Agree nor Disagree	Disagree	Strongly Disagree
○	○	○	○	○

Q176

The use of animals such as rabbits for testing the safety of cosmetics and household products is unnecessary and should be stopped.

Strongly Agree	Agree	Neither Agree nor Disagree	Disagree	Strongly Disagree
○	○	○	○	○

INdirect_brief Randomized Block Options

Q220

Now, please tell us how much you think ***THE AVERAGE AMERICAN*** agrees or disagrees with the following statements.

Q221

The average American thinks it is morally wrong to hunt wild animals just for sport.

Strongly Agree	Agree	Neither Agree nor Disagree	Disagree	Strongly Disagree
○	○	○	○	○

Q222

The average American does not think that there is anything wrong with using animals in medical research.

Strongly Agree	Agree	Neither Agree nor Disagree	Disagree	Strongly Disagree
○	○	○	○	○

Q223

The average American thinks it is perfectly acceptable for cattle and hogs to be raised for human consumption.

Strongly Agree	Agree	Neither Agree nor Disagree	Disagree	Strongly Disagree
○	○	○	○	○

Q224

The average American thinks, basically, humans have the right to use animals as we see fit.

Strongly Agree	Agree	Neither Agree nor Disagree	Disagree	Strongly Disagree
○	○	○	○	○

Q225

The average American thinks the slaughter of whales and dolphins should be immediately stopped even if it means some people will be put out of work.

Strongly Agree	Agree	Neither Agree nor Disagree	Disagree	Strongly Disagree
○	○	○	○	○

Q226

The average American sometimes gets upset when they see wild animals in cages at zoos.

Strongly Agree	Agree	Neither Agree nor Disagree	Disagree	Strongly Disagree
○	○	○	○	○

Q227

The average American thinks breeding animals for their skins is a legitimate use of animals.

Strongly Agree	Agree	Neither Agree nor Disagree	Disagree	Strongly Disagree
○	○	○	○	○

Q228

The average American thinks some aspects of biology can only be learned through dissecting preserved animals such as cats.

Strongly Agree	Agree	Neither Agree nor Disagree	Disagree	Strongly Disagree
○	○	○	○	○

Q229

The average American thinks it is unethical to breed purebred dogs for pets when millions of dogs are killed in animal shelters each year.

Strongly Agree	Agree	Neither Agree nor Disagree	Disagree	Strongly Disagree
○	○	○	○	○

Q230

The average American thinks the use of animals such as rabbits for testing the safety of cosmetics and household products is unnecessary and should be stopped.

Strongly Agree	Agree	Neither Agree nor Disagree	Disagree	Strongly Disagree
○	○	○	○	○

Demo

Block Options

Q5

Age

Q7

Gender

- ○ Male
- ○ Female

Q71

Please indicate the answer that includes your entire household income (previous year) before taxes.

- Less than $25,000
- $25,000-$34,999
- $35,000-$49,999
- $50,000-$74,999
- $75,000-$99,999
- $100,000-$149,999
- $150,000 or more

Q69

What is the highest level of school you have completed or the highest degree you have received?

- Less than high school degree
- High school graduate (high school diploma or equivalent including GED)
- Some college but no degree
- Associate degree in college (2-year)
- Bachelor's degree in college (4-year)
- Master's degree
- Doctoral degree
- Professional degree (JD, MD)

Q67

Which of the following best describes your race/ethnicity?

- White, Caucasian
- Black, African American
- Asian, Pacific Islander
- Mexican, Latino
- American Indian
- Other

Q81

Are you currently married?

- Yes
- No

Q82

How many children (18 years old or younger) live in your household?

Q57

Overall, how would you characterize your political views?

Very liberal	Liberal	Somewhat liberal	Centrist	Somewhat conservative	Conservative	Very conservative
○	○	○	○	○	○	○

Q15

Which of the following best describes the area where you have lived most of your life?

- Urban
- Suburban
- Rural

Q84

Do you currently have any pets?

- Yes
- No

Q29

How important is religion in your life?

Not at all important	Not too important	Somewhat important	Very important
○	○	○	○

Q80

Do you believe in God or a universal spirit?

- Yes
- No

Q13

Do you consider yourself a vegetarian or vegan?

- Yes
- No

Add Block

End of Survey

Survey Termination Options...
